# Enhancement of Haloperidol Binding Affinity to Dopamine Receptor via Forming a Charge-Transfer Complex with Picric Acid and 7,7,8,8-Tetracyanoquinodimethane for Improvement of the Antipsychotic Efficacy

**DOI:** 10.3390/molecules27103295

**Published:** 2022-05-20

**Authors:** Abdulhakeem S. Alamri, Majid Alhomrani, Walaa F. Alsanie, Hussain Alyami, Sonam Shakya, Hamza Habeeballah, Abdulwahab Alamri, Omar Alzahrani, Ahmed S. Alzahrani, Heba A. Alkhatabi, Raed I. Felimban, Abdulhameed Abdullah Alhabeeb, Bassem M. Raafat, Moamen S. Refat, Ahmed Gaber

**Affiliations:** 1 Department of Clinical Laboratories Sciences, The Faculty of Applied Medical Sciences, Taif University, P.O. Box 11099, Taif 21944, Saudi Arabia; a.alamri@tu.edu.sa (A.S.A.); m.alhomrani@tu.edu.sa (M.A.); w.alsanie@tu.edu.sa (W.F.A.); 2 Centre of Biomedical Sciences Research (CBSR), Deanship of Scientific Research, Taif University, P.O. Box 11099, Taif 21944, Saudi Arabia; a.s.zahrani@tu.edu.sa; 3 College of Medicine, Taif University, P.O. Box 11099, Taif 21944, Saudi Arabia; hmyami@tu.edu.sa; 4 Department of Chemistry, Faculty of Science, Aligarh Muslim University, Aligarh 202002, India; sonamshakya08@gmail.com; 5 Department of Medical Laboratory Technology, Faculty of Applied Medical Sciences in Rabigh, King Abdulaziz University, Jeddah 21589, Saudi Arabia; hhabeeballah@kau.edu.sa; 6 Department of Pharmacology and Toxicology, College of Pharmacy, University of Hail, Hail 55211, Saudi Arabia; a.alamry@uoh.edu.sa; 7 School of Health and Biomedical Sciences, RMIT University, Melbourne 3001, Australia; o_s_z@hotmail.com; 8 Department of Medical Laboratory Sciences, Faculty of Applied Medical Sciences, King Abdulaziz University, Jeddah 21589, Saudi Arabia; halkhattabi@kau.edu.sa (H.A.A.); faraed@kau.edu.sa (R.I.F.); 9 Center of Excellence in Genomic Medicine Research (CEGMR), King Abdulaziz University, Jeddah 21589, Saudi Arabia; 10 Hematology Research Unit, King Fahd Medical Research Centre, King Abdulaziz University, Jeddah 21589, Saudi Arabia; 11 3D Bioprinting Unit, Center of Innovation in Personalized Medicine (CIPM), King Abdulaziz University, Jeddah 21589, Saudi Arabia; 12 National Centre for Mental Health Promotion, P.O. Box 95459, Riyadh 11525, Saudi Arabia; aalhabeeb@ncmh.org.sa; 13 Department of Radiological Sciences, College of Applied Medical Sciences, Taif University, P.O. Box 11099, Taif 21944, Saudi Arabia; bassemraafat@tu.edu.sa; 14 Department of Chemistry, College of Science, Taif University, P.O. Box 11099, Taif 21944, Saudi Arabia; 15 Department of Biology, College of Science, Taif University, P.O. Box 11099, Taif 21944, Saudi Arabia

**Keywords:** charge transfer, haloperidol, π-acceptors, antipsychotics

## Abstract

Haloperidol (HPL) is a typical antipsychotic drug used to treat acute psychotic conditions, delirium, and schizophrenia. Solid charge transfer (CT) products of HPL with 7,7,8,8-tetracyanoquinodimethane (TCNQ) and picric acid (PA) have not been reported till date. Therefore, we conducted this study to investigate the donor–acceptor CT interactions between HPL (donor) and TCNQ and PA (π-acceptors) in liquid and solid states. The complete spectroscopic and analytical analyses deduced that the stoichiometry of these synthesized complexes was 1:1 molar ratio. Molecular docking calculations were performed for HPL as a donor and the resulting CT complexes with TCNQ and PA as acceptors with two protein receptors, serotonin and dopamine, to study the comparative interactions among them, as they are important neurotransmitters that play a large role in mental health. A molecular dynamics simulation was ran for 100 ns with the output from AutoDock Vina to refine docking results and better examine the molecular processes of receptor–ligand interactions. When compared to the reactant donor, the CT complex [(HPL)(TCNQ)] interacted with serotonin and dopamine more efficiently than HPL only. CT complex [(HPL)(TCNQ)] with dopamine (CTtD) showed the greatest binding energy value among all. Additionally, CTtD complex established more a stable interaction with dopamine than HPL–dopamine.

## 1. Introduction

Atypical antipsychotics or serotonin–dopaminergic antagonists, the fourth class of antipsychotic drugs, can improve the so-called positive symptoms of schizophrenia, such as hallucinations, delusions, and agitation, as well as negative symptoms, such as catatonia and flattening of the ability to feel emotion. Each agent in this group has a unique profile of receptor interactions. Almost all antipsychotics block dopamine receptors and reduce dopamine transmission in the forebrain. Moreover, atypical antipsychotics have an affinity for serotonin receptors. Atypical antipsychotics are related to chlorpromazine and haloperidol (HPL), and HPL is used to treat acute psychotic conditions, delirium, and schizophrenia [1]. 

The formation of highly colored charge-transfer (CT) complexes that absorb light in the visible region is often related with molecular interactions between electron donors and acceptors [2,3]. CT complexes have become more important in the fields of drug receptor binding; DNA binding; and antibacterial, antifungal, and anticancer applications [4,5]. A weak interaction between donors and acceptors causes the reaction [6]. 

Donor–acceptor complexation plays an important role especially in the field of biochemical energy transfer process [7]. The formation of brilliantly colored CT complexes that absorb visible light is frequently linked to charge transfer interactions between electron acceptors and donors [2]. In biological systems, mechanisms requiring molecular complexation and structural recognition include drug design, enzyme catalysis, and ion exchanges via lipophilic membranes [3,4]. Mulliken postulated that an electron transfer from a Lewis base′s π-molecular orbital to a Lewis acid′s vacant λ-molecular orbital causes the development of molecular complexes from two aromatic molecules, with the resonance between this dative structure and the no-band structure maintaining the complex.

Solid CT products of HPL with 7,7,8,8-tetracyanoquinodimethane (TCNQ) and picric acid (PA) have not been reported till date. Therefore, we conducted this study to investigate such reactions. The molecular docking software AutoDock Vina was used to investigate the interactions between ligands (HPL and synthesized CT complexes) and receptors (serotonin and dopamine). Hydrophobic, ionizability, aromatic, and hydrogen bond surfaces were studied as well as binding energy. The best molecular docking data were submitted to molecular dynamic simulation at 300 K for 100 ns to give a more effective mechanism for illustrating receptor–ligand interactions. In terms of residue flexibility, structural stability, solvent accessible surface area, structure compactness, and hydrogen bond interactions, the dynamic properties of the complexes were compared.

## 2. Results and Discussion

### 2.1. Preface

**Figure 1:**
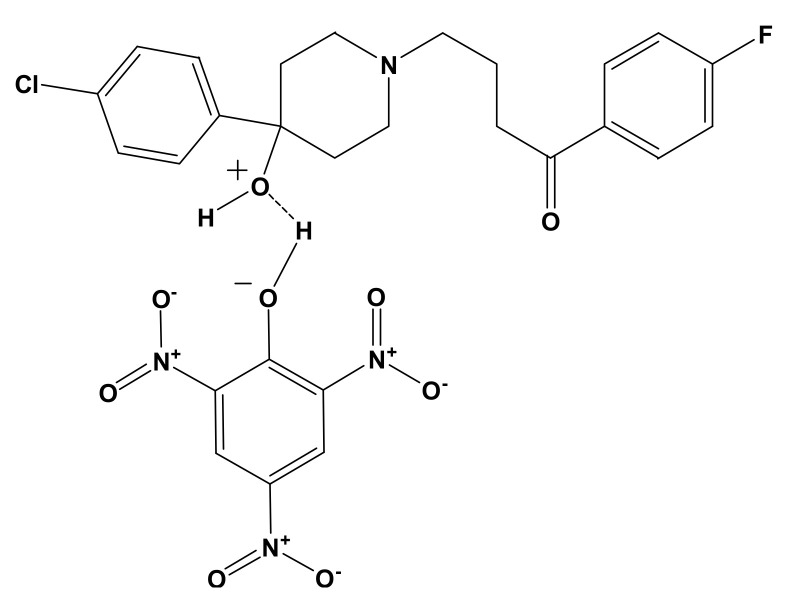
Charge-transfer (CT) complex of [(HPL)(PA)].

**Figure 2:**
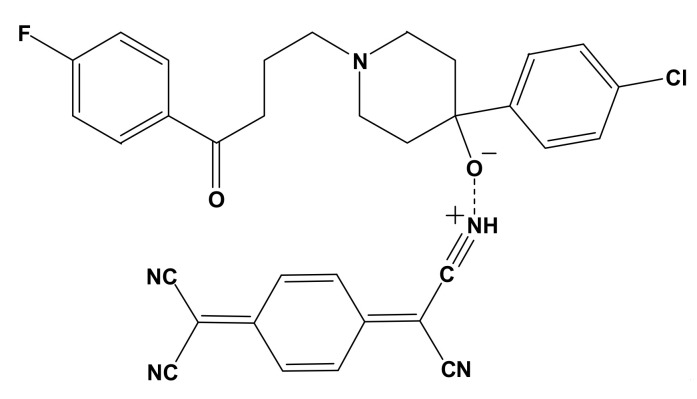
CT complex of [(HPL)(TCNQ)].

The micro analytical technique confirmed that the molar ratio between HPL donor and PA and TCNQ (π–acceptors) was 1:1. The conductivities of HPL-PA and HPL-TCNQ CT complexes were 45 and 53 Ω^−1^ cm^−1^ mol^−1^, respectively. The low conductance values of the synthesized CT complexes deduced the formation of D^+^ and A^−^ datives anions based on the association of donor–acceptor chelation. The electronic spectra of synthesized CT complexes of HPL-PA and HPL-TCNQ refer to the association of new electronic absorption bands (447 nm, 738, and 837 nm), which did not exist in the spectra of free reactants. The infrared spectrum of HPL-PA complex was assigned upon intermolecular hydrogen bonding between the –OH group of the PA acceptor and basic oxygen atom center of HPL donor (Figure 1). In the case of the infrared spectrum of HPL-TCNQ solid CT complex (Figure 2), the –OH stretching band of HPL shifted to higher frequencies. This was assigned to the increase in polarity status, –^−^O-C≡NH^+^, during the complexation process.

The bonding of the –OH group of HPL and the –OH group of PA to the –CN of TCNQ acceptors via intermolecular hydrogen bonding was confirmed by proton NMR spectra of the free HPL donor and its HPL-PA and HPL-TCNQ complexes. The activation energy (E) was used to calculate the thermal stability of both HPL-PA and HPL-TCNQ complexes using Coats–Redfern and Horowitz–Metzger techniques [8,9,10].

The average activation energies for the [(HPL)(PA)] complex and the [(HPL)(TCNQ)] complex were 132 kJ mol^−1^ and 98 kJ mol^−1^, respectively, and the variant data might be influenced by the acceptor type. The activated complexes had a more ordered structure than the reactants, and the activation of entropy (ΔS ^∗^) had negative values, indicating that the reaction rates were slower than normal.

**Figure 3:**
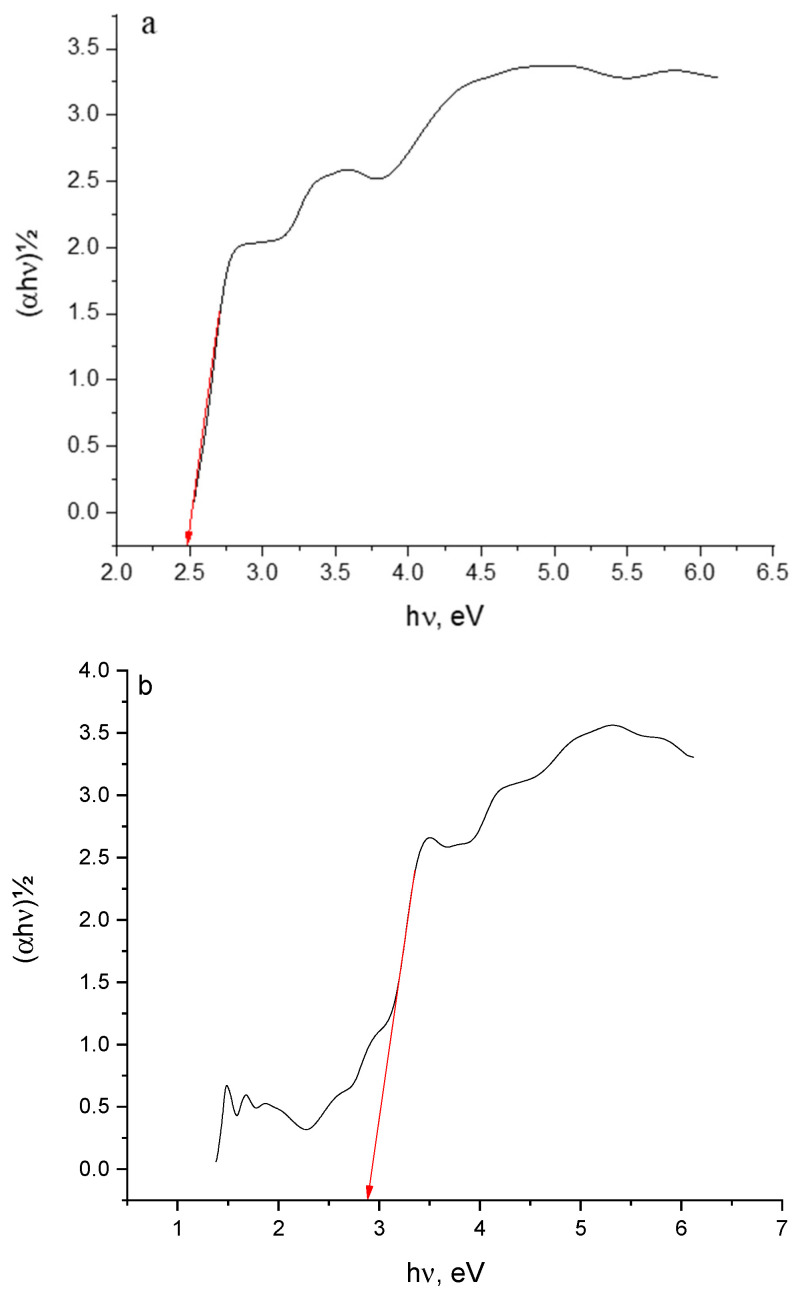
Plots of optical energy (hν) against (αhν)^½^ for (**a**) haloperidol–picric acid (HPL-PA) and (**b**) HPL-7,7,8,8-tetracyanoquinodimethane CT complexes.

The optical band gap (E_g_), which refers to the minimum transition energy, was determined based on the electronic absorption spectra. Optical absorption near the edge of the absorption band can be used to estimate E_g_ and confirm the formation of CT complexes. The absorption coefficient (α) can be estimated from the transmittance (T) of the complex according to the following equation:

α = 1/d lin (1/T)(1)

where d is the sample thickness. The bandgap of CT complexes can be calculated from the relationship between α and E_g_ based on the following equation [11]:

αhν = A(hν − E_g_)^m^ (2)

where (m) equals to ½ and 2 for direct and indirect transitions, respectively, whereas (A) is an energy-independent constant. The values of (αhν)2 were plotted against hν. The direct optical bandgap Eg was determined from the linear relationship of the plots at the absorption edge where (αhν)2 = 0 [12]. Eg values for HPL-PA and HPL-TCNQ CT complexes were 2.483 and 2.895, respectively (Figure 3), and the values were dependent on the nature of the acceptor. These data indicate the conducting behavior of HPL-PA and HPL-TCNQ complexes [13,14].

### 2.2. Molecular Docking Studies

**Figure 4:**
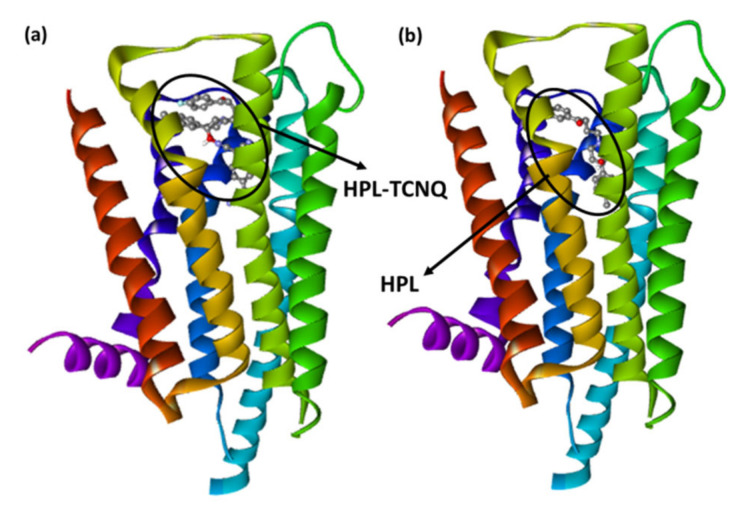
The best-docked position showing a helical model of dopamine docked with (**a**) CT complex and (**b**) HPL drug only.

The docking positions of the synthesized CT complexes [(HPL)(PA)] and [(HPL)(TCNQ)] against serotonin (PDB ID: 6A94) and dopamine (PDB ID: 6CM4) were determined. For comparison, HPL was employed as the control. CT complexes have a larger potential binding energy than HPL in both receptors (Table 1 and Table 2). Among them, [(HPL)(TCNQ)] had the greatest docking energy. The theoretical binding energies of [(HPL)(TCNQ)] with serotonin and dopamine were −10.2, and −11.8 kcal/mol, respectively. Additionally, the higher binding energy value of [(HPL)(TCNQ)]–dopamine (CTtD) signifies a stronger interaction with dopamine compared to that with serotonin. The best docking position of CTtD is shown in Figure 4, and the docking data are given in Table 2.

**Figure 5:**
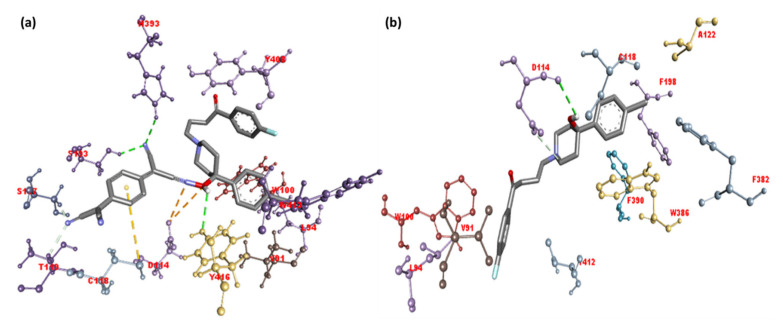
3D representation of the interactions for dopamine docked with (**a**) CT complex and (**b**) HPL drug only.

The illustration of molecular docking for ligand–receptor interactions depicted in Figure 5a,b. As shown in Figure 5a, CT complex [(HPL)(TCNQ)] with dopamine (CTtD) revealed that the amino acid residues, including His393, Ser193, and Tyr416, formed hydrogen bond interactions. Additionally, Val91 and Trp413 (π-Alkyl); Tyr408 (π-π T-Shaped); Cys118 (π-Alkyl); Thr412 and Leu94 (π-Sigma); and Asp114 (Attractive charge) interactions were present [15,16].

**Figure 6:**
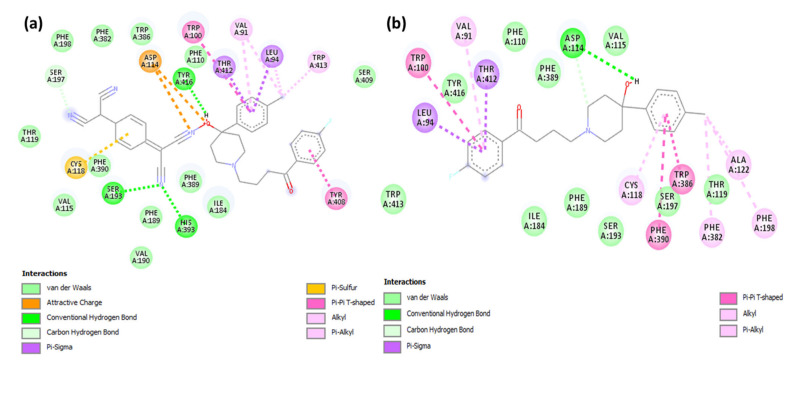
2D representation of the interactions for dopamine docked with (**a**) CT complex and (**b**) HPL drug only.

**Figure 7:**
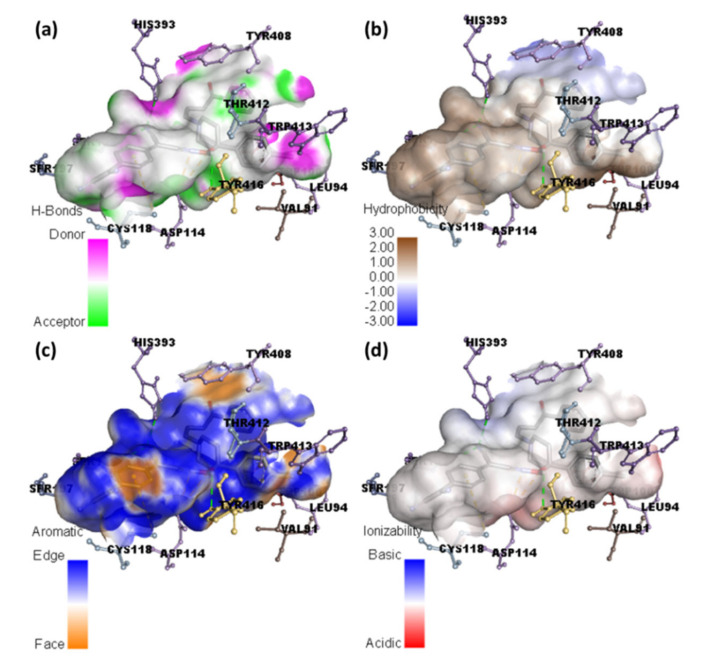
Representation of (**a**) hydrogen binding, (**b**) hydrophobic, (**c**) aromatic, and (**d**) ionizability surfaces between dopamine and CT complex.

**Figure 8:**
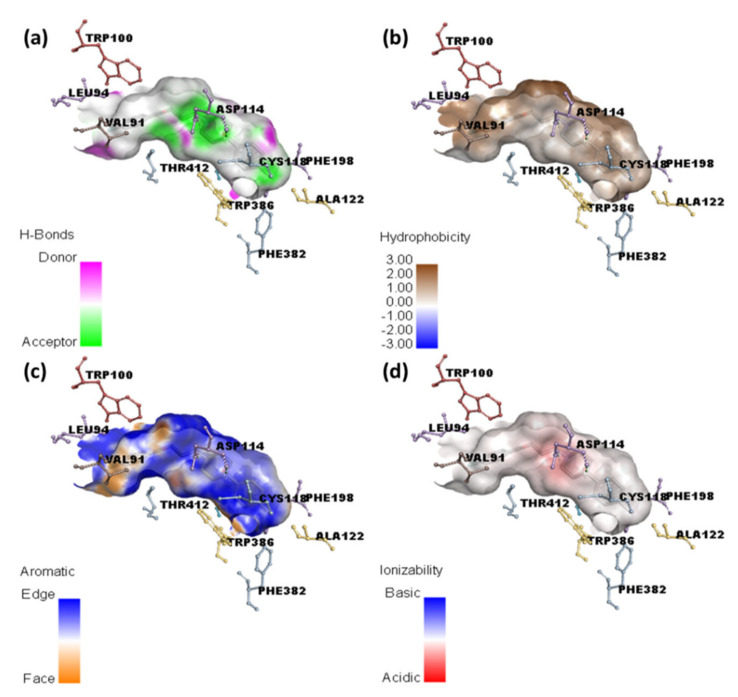
Representation of (**a**) hydrogen binding, (**b**) hydrophobic, (**c**) aromatic, and (**d**) ionizability surfaces between dopamine and HPL drug only.

Molecular docking of HPL drug with serotonin and dopamine revealed the potential binding energies as −10.0 and −10.9 kcal/mol, respectively. The higher binding energy value of HPL–dopamine (HPLD) signifies stronger interaction with dopamine compared to that with serotonin. The best docking position with dopamine (HPLD) is shown in Figure 4, and the docking data are shown in Table 2. Figure 5b shows the interaction between HPL and dopamine, which reveals that the amino acid residue Asp114 formed hydrogen bond interactions. Additionally, Phe198, Phe382, Cys118, and Val91 (π-Alkyl); Trp100, Trp386, and Phe390 (π-π T-shaped); and Lue94 and Thr412 (π-Sigma) interactions were present. This shows that the CT complex [(HPL)(TCNQ)] binds to both receptors more efficiently as compared toHPL alone, and among them, CTtD had the highest binding energy value. 2D representations of ligand–receptor interactions are shown in Figure 6. Hydrophobic, ionizability, aromatic, and hydrogen bond surfaces at the interaction site of [(HPL)(TCNQ)] and dopamine are represented in Figure 7, and those for HPL and dopamine are shown in Figure 8.

### 2.3. Molecular Dynamics Simulation

**Figure 9:**
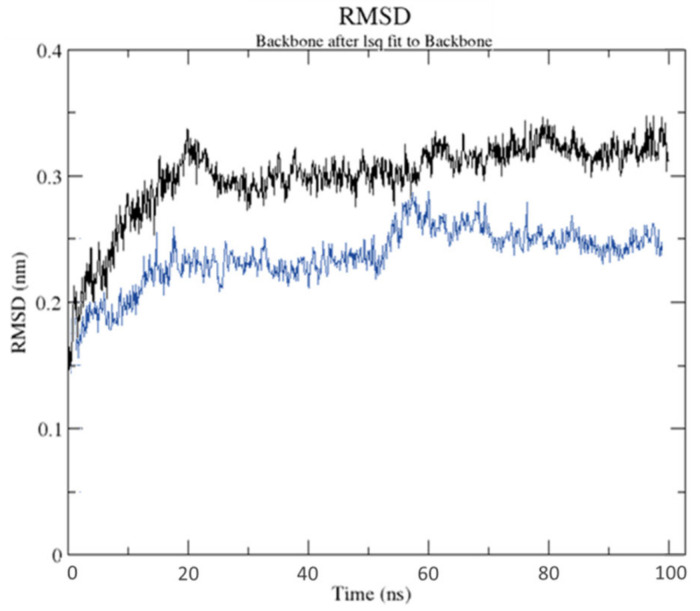
Root mean square deviation of solvated receptor backbone and ligand complex during the 100 ns MD simulation [HPLD complex (black) and CTtD complex (blue)].

The best docked location of HPLD and CTtD with the highest docking score at 100 ns molecular dynamics (MD) generated by AutoDock Vina was used. Only the best docking output was used to build up this method in a high-throughput manner for analyzing the binding mechanism of the ligand at the active site of protein under clearly defined aqueous conditions. The root mean square deviation was computed to determine structural stability from MD data (RMSD). HPLD and CTtD formed stable conformation after ~80 ns and ~65 ns, respectively, with an appropriate RMSD value of 3.04 and 2.41 Å, respectively, as seen in the RMSD plot (Figure 9).

**Figure 10:**
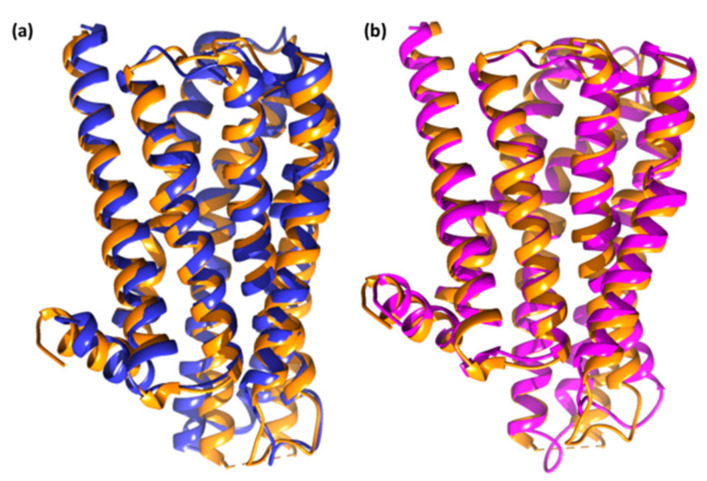
Superimposed structures of (**a**) unbounded dopamine receptor (orange) and dopamine receptor after simulation (blue) for HPLD and (**b**) unbounded dopamine receptor (orange) and dopamine receptor after simulation (pink) for CTtD.

The RMSD value range of <3.0 Å is the most acceptable [17]. CTtD produces a more stable combination as a result of this discovery. The MD findings of ligand–receptor interaction, as shown in Figure 10, bring protein chains closer together and close the distance between them [18]. Chimera 1.15 software was used to create the superimposed structures by employing the tool–structure comparison followed by the MatchMaker feature. Pairing uses both sequence and secondary structure to superimpose comparable structures.

**Figure 11:**
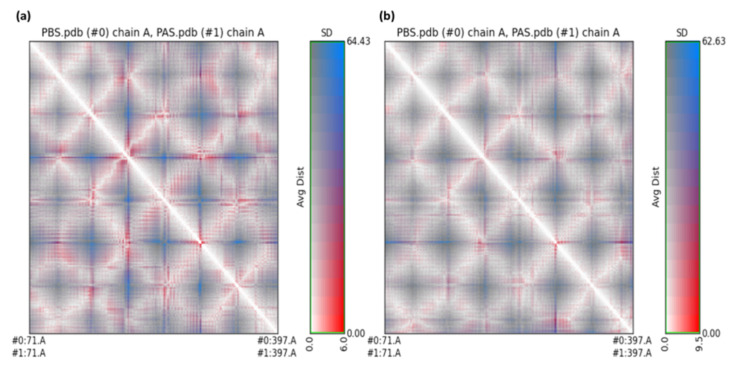
RR distance map: (**a**) unbounded dopamine receptor and dopamine receptor after simulation for HPLD and (**b**) unbounded dopamine receptor and dopamine receptor after simulation for CTtD, [PAS (#1) value = protein after simulation, SD = Standard deviation].

RR Distance Maps creates a distance map through Chimera 1.15 software by a structural comparison tool. The map can display Cα-Cα distances within a single protein chain, as well as averages and standard deviations for many chains. The white diagonal on the map represents no distance between the two residues, but the red and blue on the map reflect residue pairings with the biggest distance differences between the two conformations (Figure 11).

**Figure 12:**
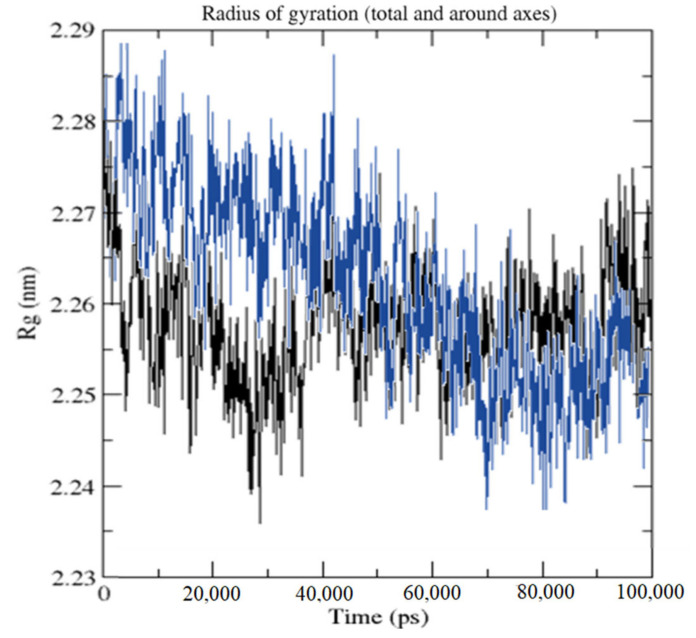
Radius of gyration for HPLD (black) and CTtD complexes (blue) during the 100-ns simulation time.

The average radius of gyration (Rg) values for HPLD and CTtD were 22.647 and 22.586 Å, respectively. Along the simulation time, Rg decreased, indicating that the structures became more compact (Figure 12).

**Figure 13:**
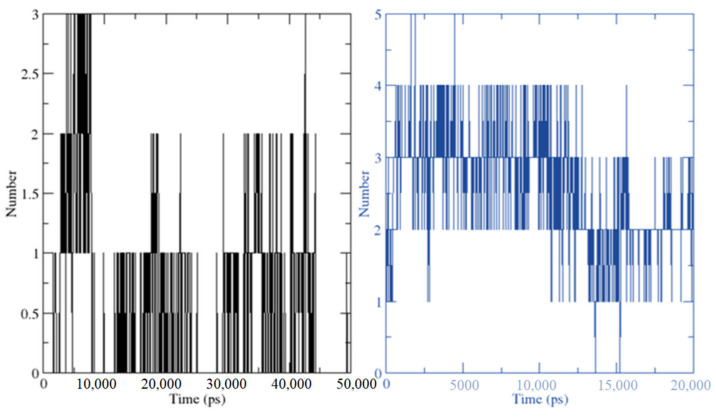
Number of average hydrogen bonding interactions between (left) HPLD complex and (right) **CTtD** complex during the 100 ns simulation time.

The number of hydrogen bond interactions between HPLD and CTtD was displayed against time using a grid-search on a 27 × 18 × 21 grid with rcut = 0.35 (Figure 13). There were 396 atoms of donors and 753 atoms of acceptors detected when the hydrogen bonds were between the ligand at 36 and 53 atoms for HPL and CT complex, respectively, and 2941 atoms of the dopamine receptor were calculated. Out of a total of 201,657 potential bonds, the average number of hydrogen bonds per period for HPLD and CTtD was 1.738 and 2.481, respectively.

Overall, the receptor–protein interaction enhanced the number of hydrogen bonds substantially, and it was more in CTtD.

**Figure 14:**
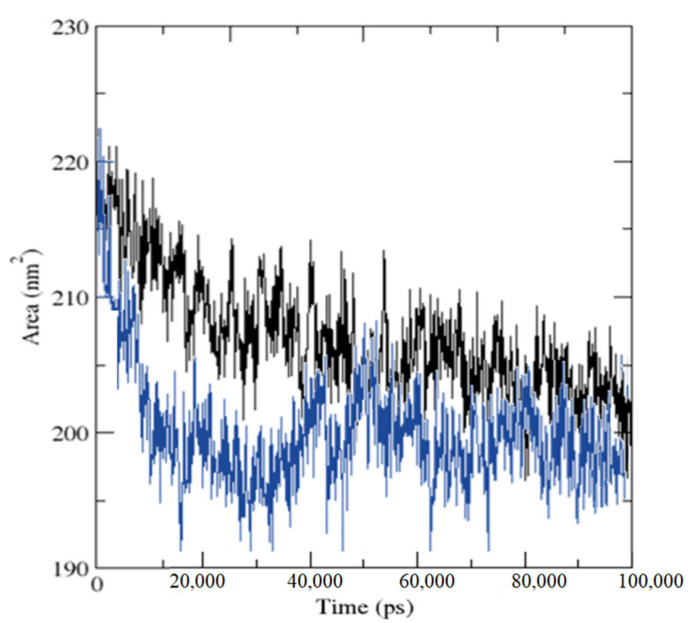
Solvent-accessible surface area analysis for HPLD complex (black) and CTtD complex (blue) during the 100 ns simulation time.

The values of solvent accessible surface area (SASA) altered as the ligand bound to the receptor (Figure 14). When the receptor binds to a ligand, the SASA value drops, indicating a change in conformation in the protein structure and a smaller pocket with more hydrophobicity around it.

## 3. Materials and Methods

### 3.1. Synthesis of [(HPL)(PA)] and [(HPL)(TCN)] CT Complexes

Previously, synthesis and characterizations (elemental analyses, conductivities, electronic absorption spectra, infrared spectra, Raman laser spectra, 1H-NMR, DSC-TG thermograms, scanning electron microscopy, energy dispersive X-ray detection, and X-ray diffraction patterns) of the two solid HPL CT complexes were performed [19]. For preparation, 3 mmol of pure HPL drugin 20 mL CH_3_OH was allowed to react with 3 mmol of each acceptor (PA and TCNQ) in 10 mL CHCl3 solvent, and both mixtures were stirred at room temperature for 45 min. Following this, the yellow and green solid complexes were isolated, washed three times with a minimum amount of CHCl3 solvent, and dried under vacuum over anhydrous CaCl_2_.

### 3.2. Physical Measurements

A Perkin–Elmer Precisely Lambda 25 UV/Vis spectrometer using a 1 cm quartz cell was used to measure the electronic absorption spectra of charge transfer complexes produced in the presence of methanol at the range of 200–800 nm. The Jenway 4010 conductivity device was also used to measure the molar conductivity in the presence of newly prepared dimethyl sulfoxide solutions. 

### 3.3. Molecular Docking

The structures of [(HPL)(PA)] and [(HPL)(TCNQ)] were obtained in PDBQT format using the OpenBabelIGUI tool (http://openbabel.org/wiki/Main Page; accessed 1 March 2022) [20]. The structure′s energy was reduced by 500 steps utilizing the MMFF94 force field and conjugation of gradient optimization procedure using PyRx-Python prescription 0.8 [21]. The RCSB protein data library was used to get the 3D crystal structures of both receptors [22]. Natural bonding and other heterogeneous atoms were removed from both acceptors by BIOVIA Discovery Studio Visualizer. The Kollman charges of the receptor were calculated, and polar hydrogen atoms were placed into the receptor using the AutoDock tool [23]. The Geistenger method was used to assign partial charges. Docking calculations were performed using AutoDock Vina [24]. The resultant docked positions were examined to check the interactions using DS Visualizer.

### 3.4. MD Simulations

Simulations were conducted using processor Intel(R) Xeon(R) CPU E5-2680 v4 @ 2.40GHz, 64 bit. MD simulation was performed using the optimal receptor–ligand complex position and an evaluation of the conformational space and inhibitory potential. MD simulation analysis with the GROMOS96 43a1 force field was performed using the Gronningen machine for chemical simulations (GROMACS, version 2019.2 package).

**Figure 15:**
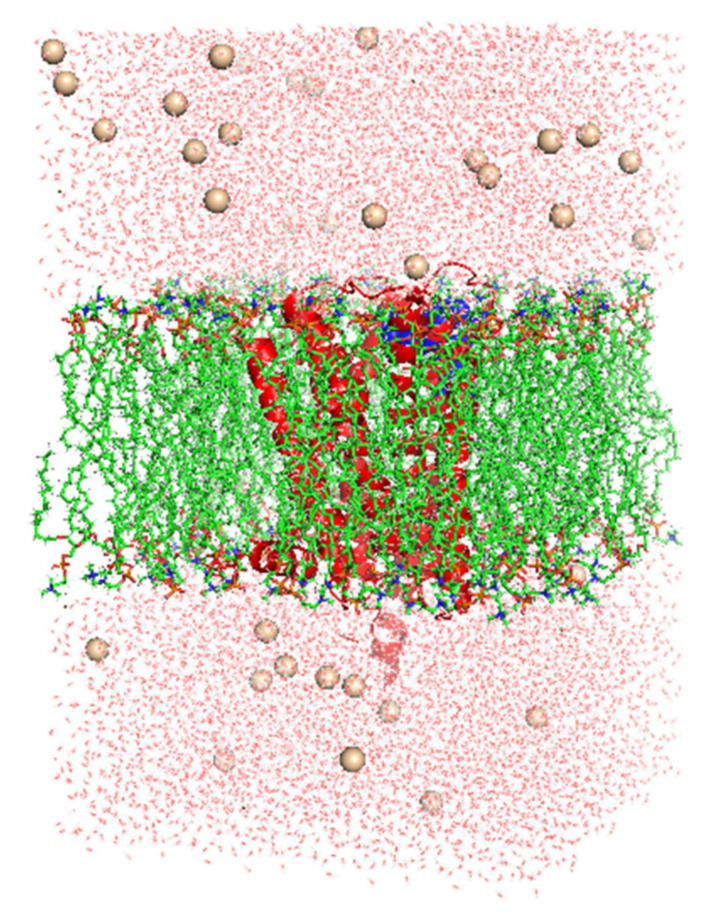
Lateral view of CTtD complex incorporated in dipalmitoylphosphatidylcholine (DPPC) membrane in a rectangular box solvated with water molecules and neutralized with 37 K^+^ and 46 Cl^−^ ions (0.15 M salt).

Both ligands′ parameter files and topologies were created using the latest CGenFF via CHARMM-GUI [25,26]. Online server CHARMM-gui was used to insert the dipalmitoylphosphatidylcholine (DPPC) membrane. Seventy two DPPC molecules were added to upperleaflet and lowerleaflet. SPC water models that extended 10 Å from the receptor were used to examine the receptor–ligand configurations in a rectangular box [27]. 37 K^+^ and 46 Cl^−^ ions (0.15 M salt) were administered to neutralize the systems and reproduce physiological salt concentrations (Figure 15).

A constant temperature (300 K) and constant pressure (1.0 bar) over 100 ns were used for simulations using the leap-frog MD complement in the NPT/NVT equilibration run [28]. The steepest descent technique with 5000 steps was also used to reduce improper contact within the system [29]. Hydrogen bonds checked with the gmx hbond tool. Rg and SASA were calculated using the gmx gyrate and gmx sasa programs, respectively. The RMSD of protein was calculated using the gmx rms tools. Trajectory examination was accomplished using GROMACS program [30]. The plots were made with Grace Software, and the visualization was done with PyMol/VMD [8,31,32].

## 4. Conclusions

In the present research, we looked into how haloperidol (HPL) interacts with two key neurotransmitters (serotonin and dopamine) that are vital in mental health. These findings were compared with the synthesized charge transfer complexes of TCNQ and PA with HPL. The [(HPL)(TCNQ)] coupled with serotonin and dopamine more efficiently than HPL alone. Also, [(HPL)(TCNQ)]–dopamine has a higher binding energy value than HPL–dopamine. The molecular dynamic simulation at 100 ns demonstrated that the [(HPL)(TCNQ)]–dopamine complex had a more stable interaction with the dopamine receptor than the HPL–dopamine complex.

## Figures and Tables

**Table 1:** Docking score of the ligands and their interactions with serotonin (6A94).

Target: PDB: 6A94			
Receptor	Binding Free Energy (kcal/Mol)	Interactions	
		H-Bond	Others
HPL-PA	−9.4	Thr160	Leu229, Phe243, Val366 (π-Alkyl); Trp336, Phe340 (π-π T-Shape)
HPL-TCNQ	−10.2	Asn363 and Tyr139	Ala321, Val324 (π-Alkyl); Ala360 (π-Alkyl); Val366 (π-Sigma)
HPL	−10.0	Leu229	Phe332, Phe243, Val366 (π-Alkyl); Phe340, Trp336, Ser159 (π-π T-Shape)

**Table 2:** Docking score of the ligands and their interactions with dopamine (6CM4).

Target: PDB: 6CM4			
Receptor	Binding Free Energy (kcal/Mol)	Interactions	
		H-Bond	Others
HPL-PA	−9.6	Trp100 and Tro419	Cys118, Val115, Leu94 (π-Alkyl); Phe390, Trp389, Tyr480 (π-π T-Shape)
HPL-TCNQ	−11.8	His393, Ser193, and Tyr416	Val91 and Trp413 (π-Alkyl); Tyr408, (π-π T-Shaped); Cys118 (π-Alkyl); Thr412 and Leu94 (π-Sigma); Asp114 (Attractive charge)
HPL	−10.9	Asp114	Phe198, Phe382, Cys118, Val91 (π-Alkyl); Trp100, Trp386, Phe390 (π-π T-shaped); Lue94, Thr412 (π-Sigma)

## Data Availability

All the data supporting reported results are available in the manuscript.

## Sample Availability

Samples of all compounds are available from the corresponding authors.
